# Dr. Lawrence Trowbridge

**Published:** 1912-04

**Authors:** 


Dr. Lawrence Trowbridge of New York, son of the late Dr. Trowbridge of Palmyra died in New York of ptomaine poisoning, March 21, 1912.

